# Anterior Palatal Radicular Cyst: A Case Report

**DOI:** 10.7759/cureus.60464

**Published:** 2024-05-16

**Authors:** Prasanna R Sonar, Aarati Panchbhai, Ankita Pathak, Aachal N Lande, Sandeep Kalisipudi, Osama Ahmed

**Affiliations:** 1 Oral Medicine and Radiology, Sharad Pawar Dental College and Hospital, Datta Meghe Institute of Higher Education and Research (DU), Wardha, IND; 2 Prosthodontics, Sharad Pawar Dental College and Hospital, Datta Meghe Institute of Higher Education and Research (DU), Wardha, IND; 3 Dentistry, Sharad Pawar Dental College and Hospital, Datta Meghe Institute of Higher Education and Research (DU), Wardha, IND; 4 Pedodontics and Preventive Dentistry, Lenora Institute of Dental Sciences, Rajanagaram, IND; 5 Dentistry, M A Rangoonwala College of Dental Sciences and Research Center, Pune, IND

**Keywords:** multidisciplinary approach, surgical enucleation, endodontic therapy, periapical lesion, odontogenic cyst, radicular cyst

## Abstract

The most prevalent kind of odontogenic cysts is radicular cysts, which usually develop from the epithelial remnants in the periodontal ligament as a result of inflammation that follows pulp necrosis. We report a case of a 49-year-old male patient who complained of painless swelling in the maxillary anterior region, which turned out to be a radicular cyst. Upon clinical examination, a soft, nontender swelling that fluctuated was found. A periapical lesion was found upon radiographic assessment. A radicular cyst was tentatively diagnosed based on clinical and radiological features. The treatment plan included enucleation, restoration of the defect with bone graft, and endodontic therapy with antibiotics. Endodontic therapy was administered after the cystic lesion was surgically removed. The diagnosis of a radicular cyst was validated by histopathological analysis. The significance of a multidisciplinary approach for the successful management of radicular cysts is emphasized in this case report, which also underscores the need for a comprehensive clinical and radiographic evaluation for accurate diagnosis. Prompt identification and suitable intervention are essential to avert possible complications and guarantee successful treatment results.

## Introduction

Any diseased cavity that is filled with fluid or semifluid material but not pus is called a cyst [1,2]. It may or may not be bordered with epithelium. Depending on how they form, cysts are either odontogenic or developmental. According to the World Health Organization's categorization, radicular cysts have an odontogenic origin [2]. The frequent inflammatory odontogenic cyst known as a radicular cyst develops from pulpal necrosis in the epithelial residues of Malassez. Radicular cysts can result from root canal infections, and while big radicular cysts greater than 1 cm in diameter are treated surgically, conventional root canal therapy is not always effective in treating radicular cysts [1-3]. The related tooth is not essential, exhibits extensive carious lesions, or has a restoration that is rarely uncomfortable or even percussion sensitive [4]. This type of cyst seldom gets big enough to entirely damage bone, and it is even less usual for it to cause the cortical plates to enlarge [5]. It is a lesion that only appears after a protracted inflammatory process that is chronic. Such a long-standing cyst occasionally experiences an extreme flare-up of the inflammatory process, quickly developing into an abscess that may eventually lead to cellulitis or form a draining fistula. Although the reason for the abrupt flare-up is unknown, loss of localized or widespread tissue resistance could be the culprit [2]. This case study demonstrates the value of a multidisciplinary approach for the successful therapy of radicular cysts and underscores the necessity of a comprehensive clinical and radiographic assessment for an accurate diagnosis.

## Case presentation

**Figure 1:**
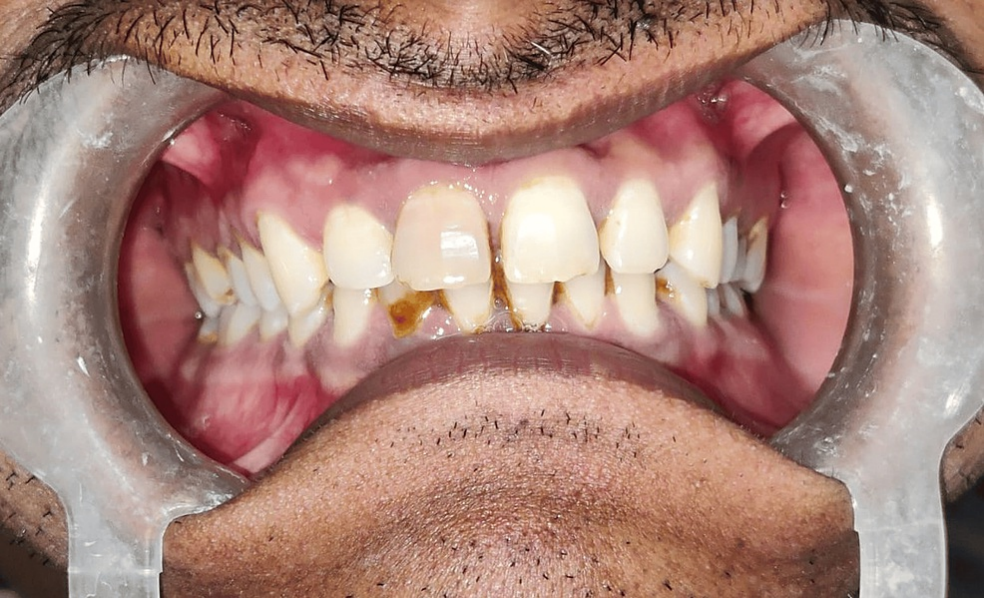
Discolored tooth 11. Image Credit: Prasanna Sonar.

**Figure 2:**
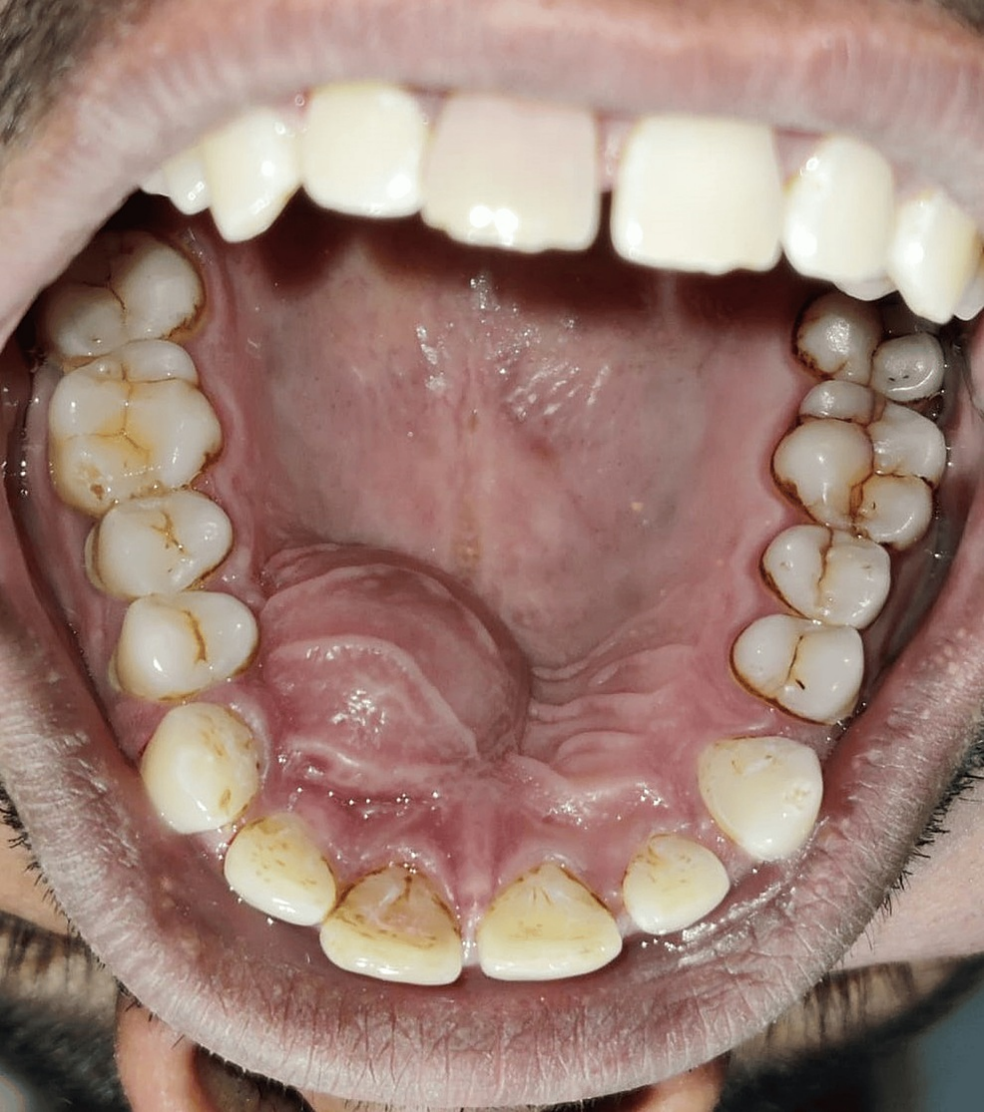
Occlusal view of dome-like swelling present palatally in relation to the 11-14 region. Image Credit: Prasanna Sonar.

The patient, 49 years old, reported eight days of painless swelling in the upper front region of the jaw. He saw a small-sized swelling over the upper anterior region of the jaw that had grown to its present size. No history of trauma was present. As it was asymptomatic swelling, there was no history of difficulty in mastication or deglutition. Also, there was no history of bleeding or pus discharge. Past medical history and past dental history were not significant; no deleterious habits were found. The patient did not report any history of trauma. Intraoral examination revealed discoloration with 11 as shown in Figure 1. A well-defined dome-like swelling was seen on the right side of the palate in relation to the 11-14 region as shown in Figure 2. No tenderness was present on vertical and lateral percussion in associated teeth. The well-defined asymptomatic swelling had a size of 2 x 2 cm and had a roughly round shape with a raised and tensed surface. The color was the same as that of adjacent mucosa. On palpation, the fluctuant swelling had a soft consistency. Mobility was not seen in any of the teeth. The clinical diagnosis was given as a periapical cyst with 11. Periapical dental abscess, periapical granuloma, residual cyst and odontogenic keratocyst were considered as clinical differential diagnosis considering anatomical site.

**Figure 3:**
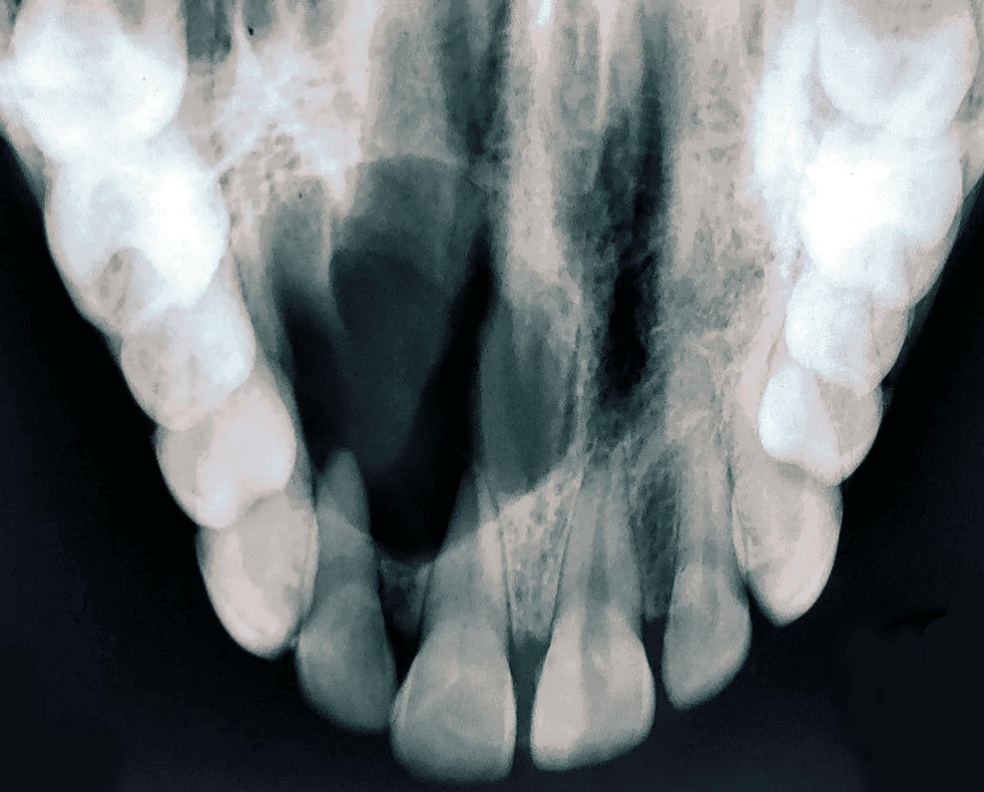
Occlusal radiograph showing the lesion in 11-14 region. Image Credit: Prasanna Sonar.

**Figure 4:**
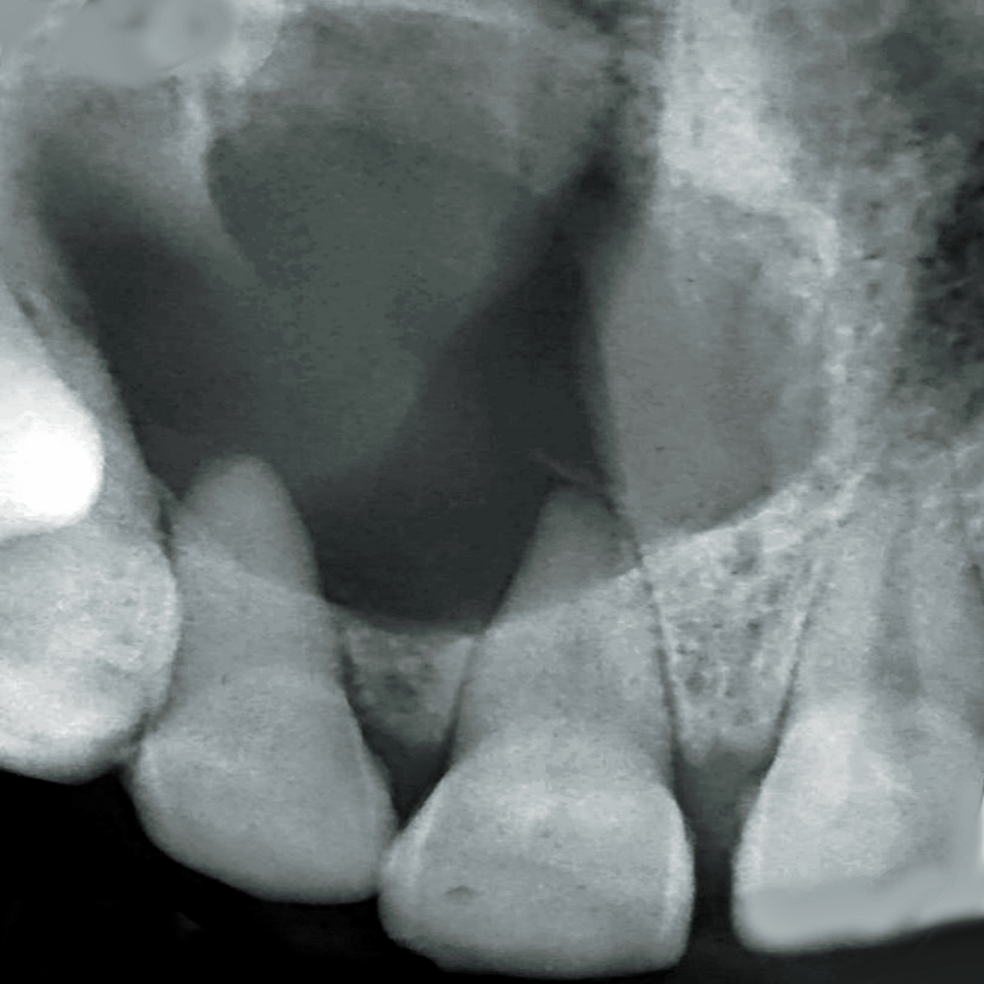
Intraoral periapical radiograph with 11-13 region. Image Credit: Prasanna Sonar.

**Figure 5:**
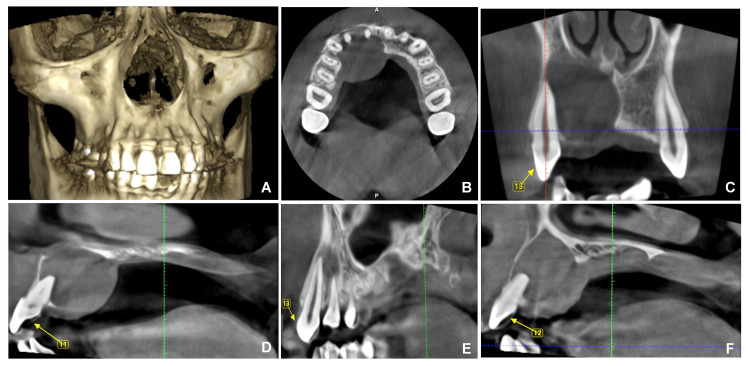
CBCT of the patient. A: Three-dimensional reconstructed image of CBCT; B: axial section; C: coronal section with 13; D: sagittal section with 11; E: sagittal section with 13; and F: sagittal section with 12. CBCT: cone-beam computed tomography. Image Credit: Prasanna Sonar.

Electric and thermal tests with 11-14 were negative. The occlusal radiograph and the intraoral periapical radiograph showed a well-defined radiolucency seen in the periapical region in relation to 11-14; the margins were well-defined and corticated. Displacement of root of 12, root resorption and wide apex with 11 were also seen. The swelling revealed a radicular cyst on radiographic examination. Figure 3 shows an occlusal radiograph. Figure 4 shows an intraoral periapical radiograph. Cone-beam computed tomography (CBCT) showed the lesion of size 20.24 x 21.35 x 20.47 mm (antero-posteriorly (A/P) x medio-laterally (M/L) x superio-inferiorly (S/I)). Root apex of 11-13 was involved in the lesion with severe bone resorption. Root resorption was seen with 11. Figures 5A-5F show CBCT of the patient. Radicular cysts typically appear at a nonvital tooth's apex. At the root tip, these cysts manifest as distinct, round to oval radiolucencies. The border of radicular cysts is usually thin and corticated. Usually, the lesion appears radiolucent and homogenous. Therefore, the clinical diagnosis of radicular cyst was considered as provisional diagnosis. Ameloblastoma, odontogenic keratocyst and periapical granuloma were given as radiological differential diagnosis.

**Figure 6:**
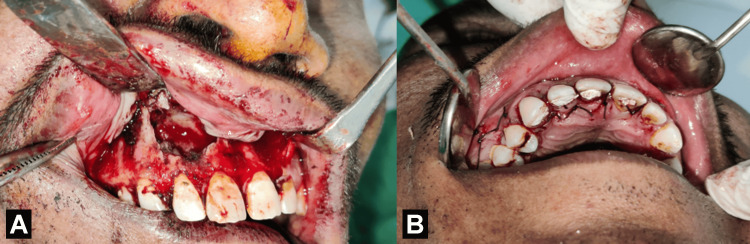
Patient's mouth with raised full-thickness mucoperiosteal flap and closure of the surgical defect. A: Raised full-thickness mucoperiosteal flap; and B: closure of the surgical defect with sutures. Image Credit: Prasanna Sonar.

**Figure 7:**
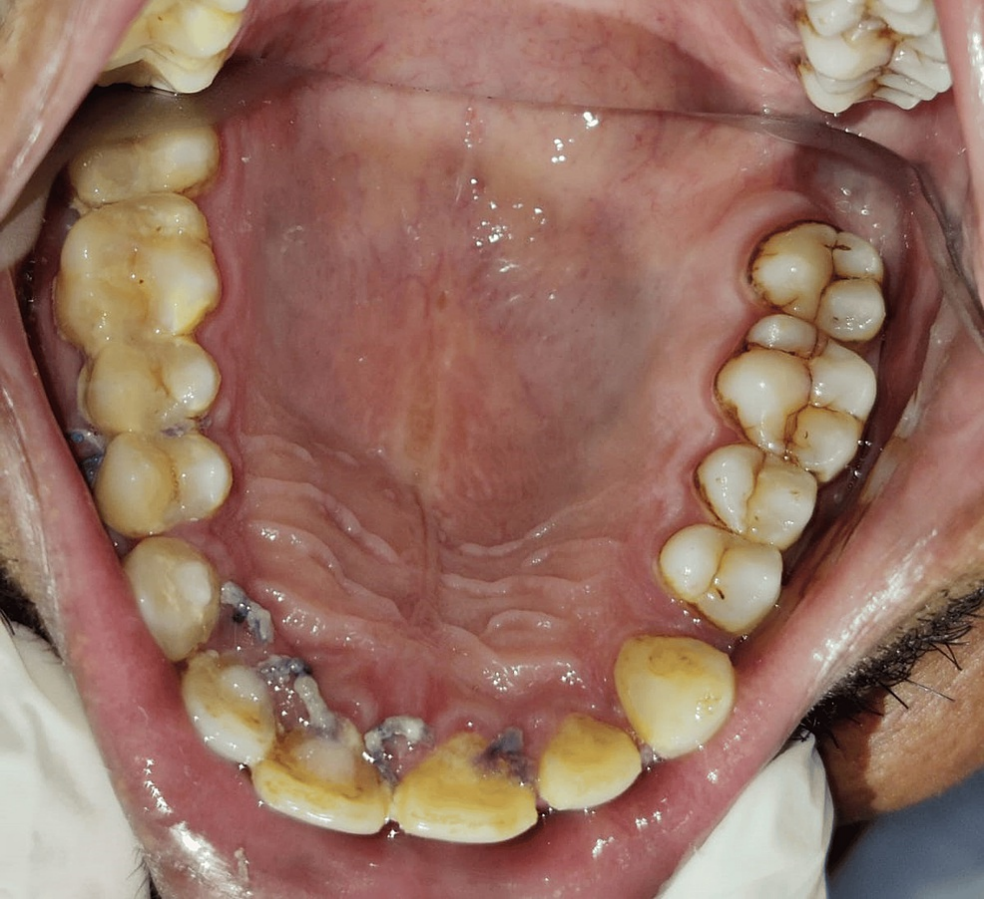
Postoperative clinical photograph after seven days. Image Credit: Prasanna Sonar.

**Figure 8:**
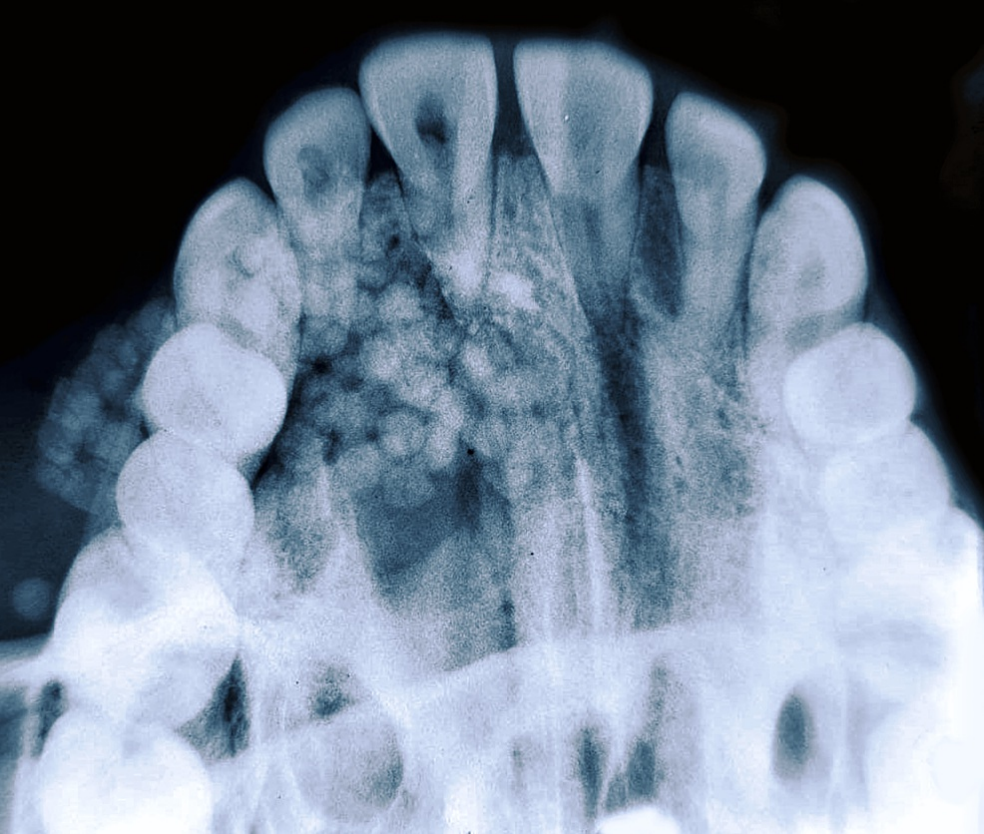
Postoperative occlusal radiograph after seven days. Image Credit: Prasanna Sonar.

**Figure 9:**
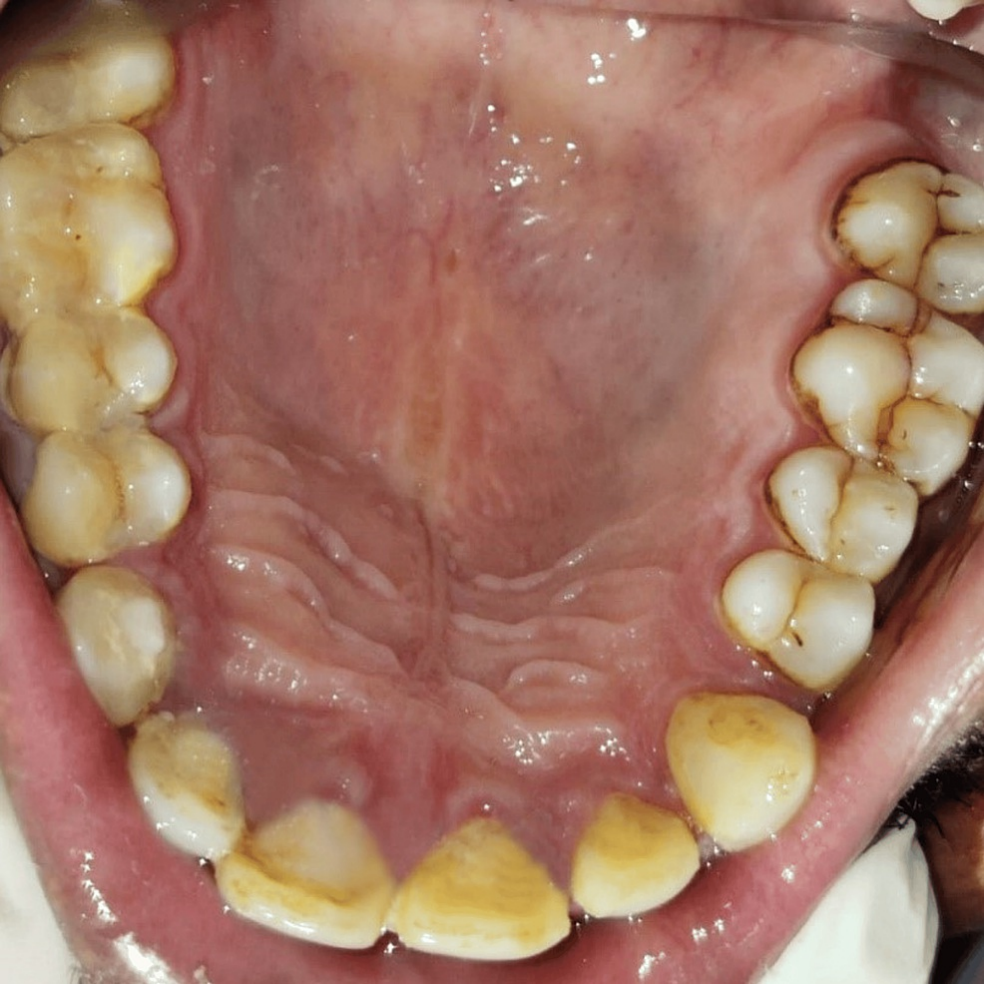
Postoperative clinical follow-up photograph after 14 days. Image Credit: Prasanna Sonar.

Following the patient's explanation of the treatment plan, his informed consent was obtained. The treatment strategy for the patient included enucleation of the lesion, repair of the defect with a freeze-dried bone allograft, endodontic treatment and a course of antibiotics. The choice of treatment plan was enucleation of the lesion guided by CBCT followed by root canal treatment. The following visit, the patient was ready for surgery, which involved surgically removing the cyst and using a bone graft to fill the deficiency. A crevicular incision was performed in the labial area 11-13 following the application of local anesthetic (Lidocaine). Clinically, there was a noticeable big bone defect after a full-thickness mucoperiosteal flap was raised. After completing curettage, the cystic lesion was enucleated, granulation tissue was removed, and the lesion was sent for histological analysis. An allograft of freeze-dried bone was used to restore the deficiency. A 3-0 silk was used to close the flap. Figure 6A illustrates the raised full-thickness mucoperiosteal flap. Figure 6B illustrates closure of the flap with sutures. The patient received postoperative instructions and was continued on antibiotics and analgesics. Endodontic therapy was finished following the cystic lesion's surgical enucleation. Root canal opening with 11-13 was completed when a rubber dam was applied. Following the biomechanical preparation and working length determination, a week-long intracanal medication of calcium hydroxide was employed. At the following appointment, the obturation was finished. Figure 7 shows a postoperative follow-up photograph after seven days. Figure 8 shows a postoperative occlusal radiograph after seven days. On histopathological examination, the thin, nonkeratinized stratified squamous epithelium revealed a cystic lining. There was an invasion of inflammatory cells, primarily plasma cells and lymphocytes, within the connective tissue capsule. Histopathological examination confirmed the diagnosis of a radicular cyst. Clinical observations revealed a significant reduction in lesion size after 14 days. Figure 9 shows a postoperative follow-up photograph after 14 days. The patient is currently satisfied and will be monitored for a year.

## Discussion

Of all the cysts affecting the jaws, radicular cysts are thought to be the most frequent, accounting for 52%-68% of cases. True cysts make up 9% of the cysts, whereas pocket cysts make up 6% [6]. Men are more likely to be afflicted than women, with the maximum prevalence occurring in the third decade of life [3]. Radicular cyst incidence in deciduous teeth appears to be lower than in permanent teeth. One explanation for this could be the primary teeth's shorter lifespan, which also makes drainage easier due to the many accessory canals present [7].

Radicular cysts caused by trauma or dental caries are regarded as inflammatory cysts [1,2,8,9]. The primary cause of radicular cysts is dental caries or trauma that results in pulpal necrosis, from which an infection spreads to the tooth's apex and forms a periapical cyst. This infection is triggered by local inflammation and results in an epithelial rest of Malassez. Three stages have been identified in the pathophysiology of radicular cysts: the beginning phase, cyst development, and expansion [9]. On the creation of cyst cavities, two ideas exist. According to the "abscess theory", cavity abscess created by tissue necrosis and lysis is lined by proliferating epithelium because epithelial cells have an inbuilt propensity to cover exposed surfaces of connective tissue, and the cyst expands by osmosis [3]. The assumption underlying the "nutritional deficiency theory" is that there is no nutritional insufficiency. Then diffuses into the cyst cavity, raising the intraluminal hydrostatic pressure much above the capillary pressure. Increased intracystic pressure can cause the cyst to enlarge and cause bone resorption. Radicular cysts may occur because of osmotic pressure, which can cause pocket cysts with lumen open to the necrotic root canal to enlarge more than typical [3].

Clinically, periapical cyst patients are asymptomatic until an acute inflammatory aggravation occurs, the cyst enlarges, and swelling ensues. It is possible to notice mild sensitivity in the region of concern. As the cyst grows, neighboring teeth may move and become mobile [2]. A distinct unilocular radiolucency situated periapical near a tooth involving pulpal involvement is revealed by a radiographic characteristic [10]. A radicular cyst's edge is radiopaque and continuous with the loss of lamina dura, with hyperostotic boundaries. On the other hand, the radiopaque edge could not be present or readily seen in diseased or quickly growing cysts. The chronic radicular cyst may cause the tooth roots to resorb [10].

Based on a histological analysis, the majority of radicular cysts have nonkeratinized-stratified squamous epithelium lining them, which can vary in thickness. The epithelial lining may exhibit proliferation and an arcading arrangement in the early stages, along with a high level of inflammatory infiltration. The lining of expanding cysts becomes quiet, rather uniform, and differentiates to a point where it resembles simple stratified squamous epithelium. Seldom does keratin production occur, and when it does, it mostly affects the cyst wall. Polymorphonuclear leukocytes make up the majority of the inflammatory cell infiltrate in the proliferative epithelium. Chronic inflammatory cells invade the surrounding fibrous capsule [11].

Rarely, metaplastic alterations in the radicular cysts' epithelial lining might give birth to squamous carcinoma [10,12,13]. Long-term examples of radicular cysts have shown histopathological evidence of a shift from a cystic lining to epithelial dysplasia and subsequent advancement as infiltrating squamous carcinomas [1,2]. Nonsurgical endodontic treatment is a viable initial treatment option for periapical lesions and cyst-like lesions. For a good prognosis, surgical management may be required in certain circumstances. Increased understanding of microbes will benefit oral and maxillofacial surgical procedures [14]. One to two years following therapy, a follow-up is required.

The lesion's extent, relationship to important structures, origin, and clinical features, as well as the patient's participation and overall health, may all influence the treatment option. Endodontic therapy is one of the conservative treatment options for these cysts that is still being discussed by many medical professionals. Nonsurgical endodontic therapy has been reported to result in a high percentage of 94.4% in complete and partial healing of small periapical lesions. However, in large lesions, endodontic treatment alone is ineffective and should be associated with decompression, marsupialization, or even enucleation of the cyst [15]. Large periapical lesions have traditionally been treated surgically; however, calcium hydroxide treatment is a more cautious nonsurgical option that should not be disregarded [16].

Owing to the lesion's size, a conservative surgical treatment was chosen. Unlike techniques like en bloc resection, which removes both the diseased tissue and the normal structure, the conservative method focuses just on removing the infected tissues. Either marsupialization or enucleation is the surgical technique used to treat cystic lesions of the jaws. The lesion's size, location, bone integrity of the cystic wall, and closeness to important structures all influence the best course of treatment [17]. The mineral trioxide aggregate (MTA) is a common filler material for root ends [18,19]. Because MTA is more biocompatible and has a better sealing capacity than the root-end filling materials currently on the market, it has been preferred [20]. In this specific case, curettage and enucleation of the lesion were performed before root canal therapy.

Calcium hydroxide is the intracanal medication in the present case. Due to its highly alkaline nature, tissue dissolving effect, ability to induce repair through hard tissue formation and bactericidal effect, calcium hydroxide has historically been widely used as an intracanal endodontic material. However, because it will remain in the tissue for a significant duration, it cannot be deemed biocompatible [21]. Its impact on bacterial cytoplasmic membranes, denaturation of proteins, damage to DNA, action on lipopolysaccharides, and other mechanisms account for its antibacterial properties [22]. Although calcium hydroxide has long been considered the safest agent, certain investigations have found that it can have harmful side effects, including bone necrosis, cytotoxicity on cell cultures, and neurotoxicity. Additionally, if the material is extruded under high pressure during endodontic treatment, several studies have documented detrimental effects. According to a few investigations, intracanal calcium hydroxide administration would promote osseous repair and promote periapical healing by directly affecting periapical inflammatory tissue by the diffusion of hydroxyl ions (OH-) through dentinal tubules [23]. It also prevents osteoclastic activity where root resorption takes place. Furthermore, it was noted in a prior study [24] that inadvertently pushing Ca (OH)_2_ paste into the periapical lesion had no negative effects, but healing might take longer. It has been discovered that calcium hydroxide can be resorbed extraradicularly without causing any harm, and it has shown to be both clinically and radiographically successful [25].

## Conclusions

To sum up, this case report clarifies the clinical manifestation, diagnostic obstacles, and treatment approaches associated with radicular cysts. A conclusive diagnosis was made by carefully reviewing the patient's clinical characteristics, history, and radiological results. A multidisciplinary strategy including endodontic therapy and surgical intervention was used for the successful care, which resulted in the resolution of symptoms and the restoration of oral health. The aforementioned instance highlights the significance of prompt discovery, precise diagnosis, and suitable treatment to avert possible consequences linked to radicular cysts. In addition, additional research and long-term monitoring are necessary to improve our comprehension of the pathophysiology and best practices for treating these lesions.

A frequent odontogenic cyst that develops from pulpal necrosis in a nonvital tooth is called a radicular cyst. In addition to causing a well-defined radiolucency around the damaged tooth's apex, it can also cause several other clinical symptoms, including pain, edema, and infection. Usually, radiographic imaging, histological analysis, and clinical examination are used in the diagnosis process. Depending on the size, location, and accompanying symptoms, alternatives for treatment include endodontic therapy, surgical enucleation, or marsupialization. To avoid complications and guarantee the best possible outcomes for patients, early detection and effective therapy are essential. Preventive steps to reduce the chance of developing radicular cysts include practicing good oral hygiene, seeing the dentist regularly, and treating tooth infections as soon as they arise. To ensure the complete and effective treatment of radicular cysts, cooperation among oral surgeons, endodontists, and other dental specialists should be encouraged. The value of educating patients about good oral hygiene habits, routine dental checkups, and the need for early intervention in the management and prevention of radicular cysts are emphasized.
